# Supplementary material for: Curbing gastrointestinal infections by defensin fragment modifications without harming commensal microbiota
Source: Commun Biol. 2021 Jan 8;4:47. doi: 10.1038/s42003-020-01582-0 (PMC7794397; doi:10.1038/s42003-020-01582-0)
Supplement: Supplementary file 2 — Supplementary Information [file 42003_2020_1582_MOESM2_ESM.pdf]

COMMUNICATIONS BIOLOGY

Supplementary Information for

# Curbing gastrointestinal infections by defensin fragment modifications without harming commensal microbiota

Louis Koeninger[1*], Lisa Osbelt[2,3], Anne Berscheid[4,5], Judith Wendler[1], Jürgen Berger[6], Katharina Hipp[6], Till R. Lesker[2], Marina C. Pils[7], Nisar P. Malek[1], Benjamin A. H. Jensen[8], Heike Brötz-Oesterhelt[4,5,9], Till Strowig[2,10#], Jan Wehkamp[1,9#]

#these authors jointly supervised this study

*Corresponding author:

Louis Koeninger

E-mail: louis.koeninger@med.uni-tuebingen.de

[1]Department of Internal Medicine I, University Hospital Tübingen, Tübingen, Germany, [2]Department of Microbial Immune Regulation, Helmholtz Centre for Infection Research, Braunschweig, Germany, [3]ESF International Graduate School on Analysis, Imaging and Modelling of Neuronal and Inflammatory Processes, Otto-von-Guericke University, Magdeburg, Germany, [4]Department for Microbial Bioactive Compounds, Interfaculty Institute of Microbiology and Infection Medicine, University of Tübingen, Tübingen, Germany, [5]German Center for Infection Research (DZIF), partner site Tübingen, [6]Max-Planck Institute for Developmental Biology, Electron Microscopy, Tübingen, Germany, [7]Mouse Pathology and Histology, Helmholtz Centre for Infection Research, Braunschweig, Germany, [8]Novo Nordisk Foundation Center for Basic Metabolic Research, Human Genomics and Metagenomics in Metabolism, Faculty of Health and Medical Sciences, University of Copenhagen, Copenhagen, Denmark, [9]Cluster of Excellence - Controlling Microbes to Fight Infections, Tübingen, Germany, [10]Cluster of Excellence - Resolving Infection Susceptibility, Hannover, Germany

## This PDF file includes:

Figures: S1 to S4

Table S1

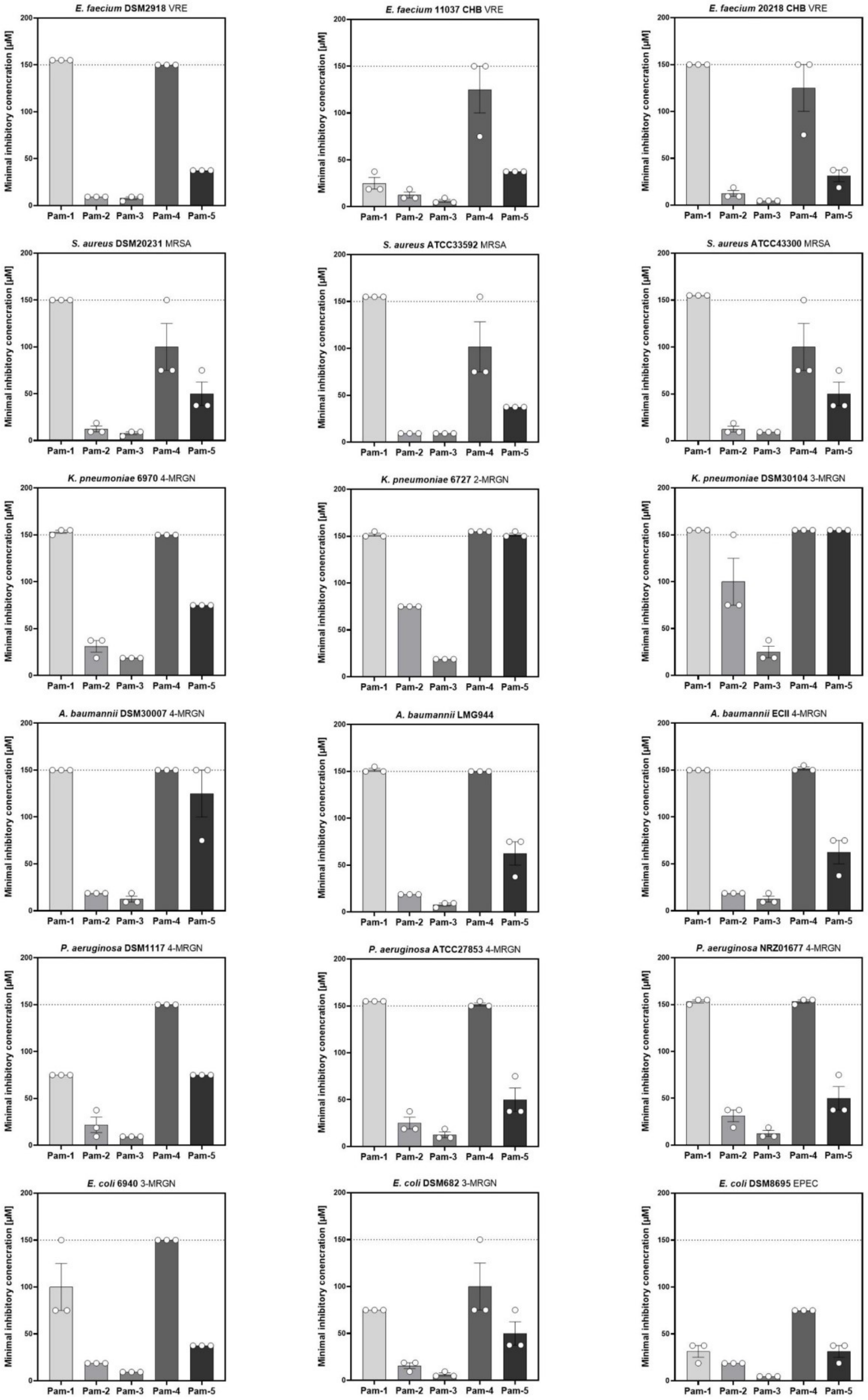


**Supplementary Figure 1. Pam's kill multidrug-resistant ESKAPE pathogens.**

Here we show the detailed results of the broth microdilution assay experiments. The dotted line marks the highest peptide concentration used in these experiments. Data are presented as mean ± SEM. Experiments were carried out three independent times.

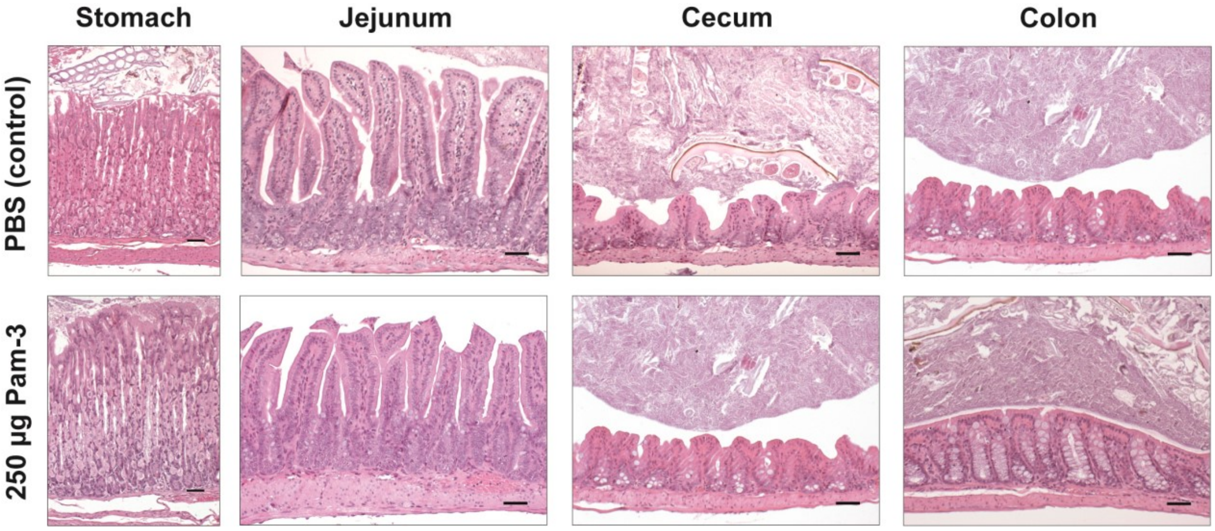


**Supplementary Figure 2. Images from the gastrointestinal tract of differentially treated mice.**

Animals were treated twice with 250 µg Pam-3 or PBS. Representative images from the stomach, small intestine (jejunum), cecum and colon are shown. Scale bars, 50 µm.

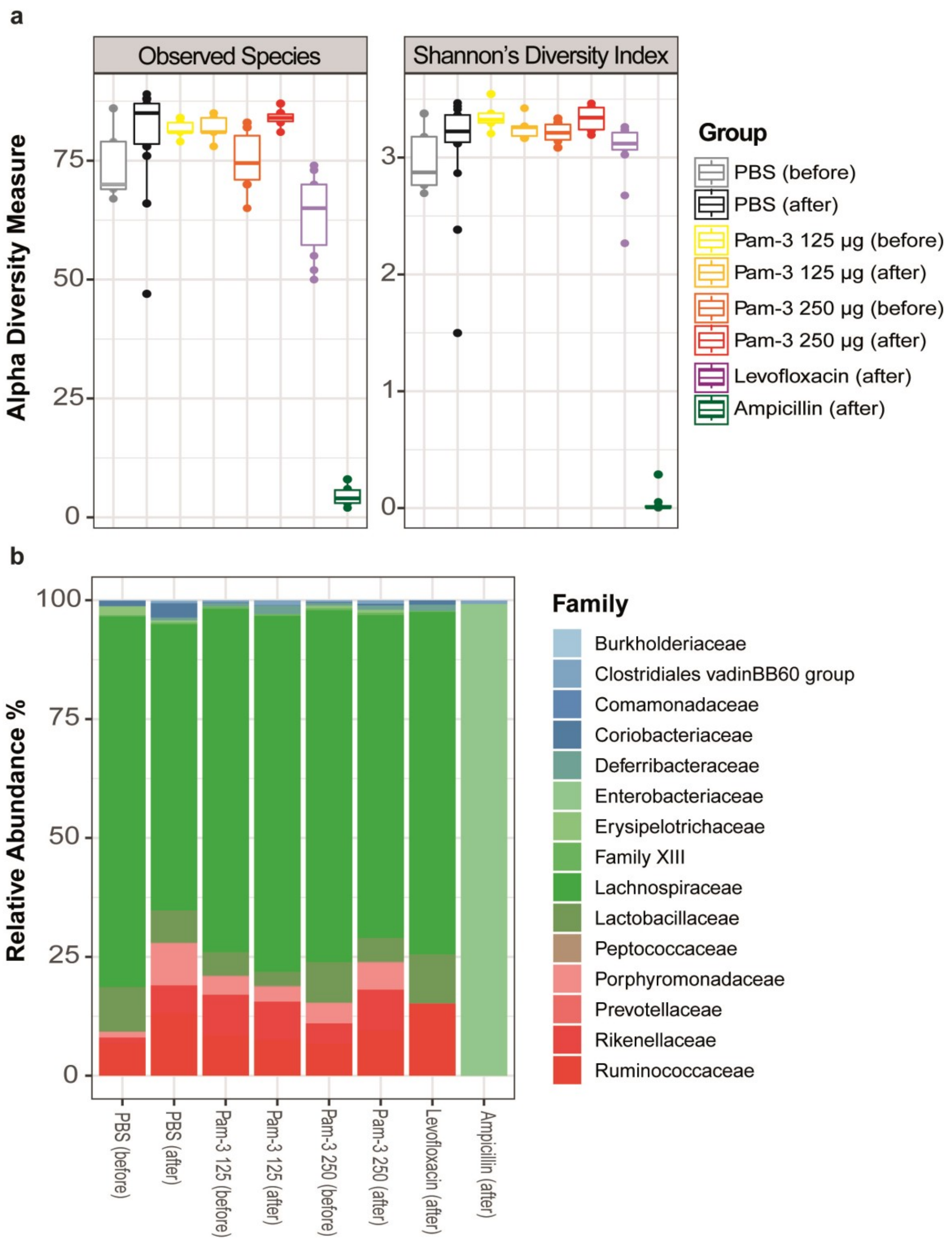


**Supplementary Figure 3. Ampicillin induces global alterations in the microbiota.**

Chow-fed mice were treated orally twice at an 8-hour interval with 125 µg Pam-3 (N = 5) or 250 µg Pam-3 (N = 6), once with Levofloxacin (N = 12) or Ampicillin (N = 10) or PBS (N = 15). (a) Feces samples were collected before and after treatment to observe changes and recovery in the microbiome. Richness (observed species) and diversity (Shannon's Diversity index) were analyzed from fecal samples. (b) Pam-3 treatment affects the abundance of bacterial genera less compared to Levofloxacin and Ampicillin.

# Analytical Data Sheet

**Name/Sequence** Pam-Ado-RGKAKCCK:

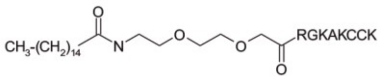


**MW** 1277

**Counter ion** TFA

**HPLC**

**Purity**
≥ 90 % (214 nm)

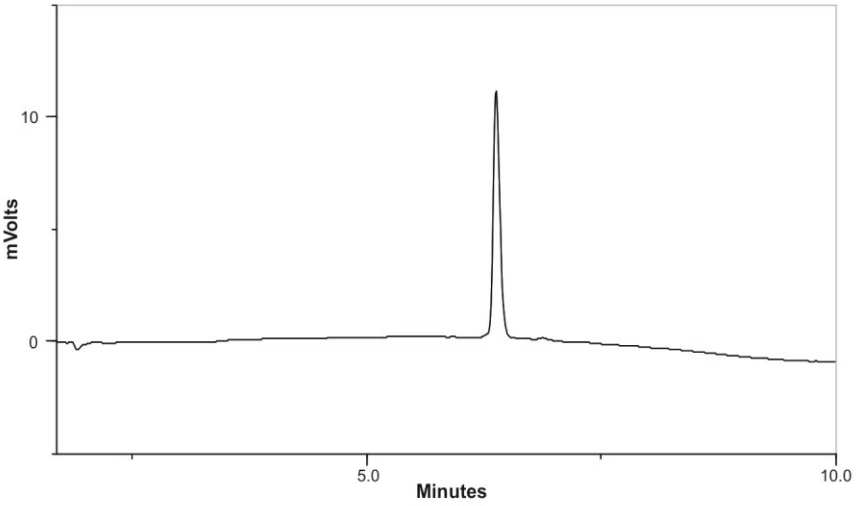


**ESI mass spectrometry**

| | |
|---|---|
| **MW (calc.)** | **1277** |
| $[M+2H]^{2+}$ | 639 |
| $[M+3H]^{3+}$ | 427 |
| $[M+4H]^{4+}$ | 320 |

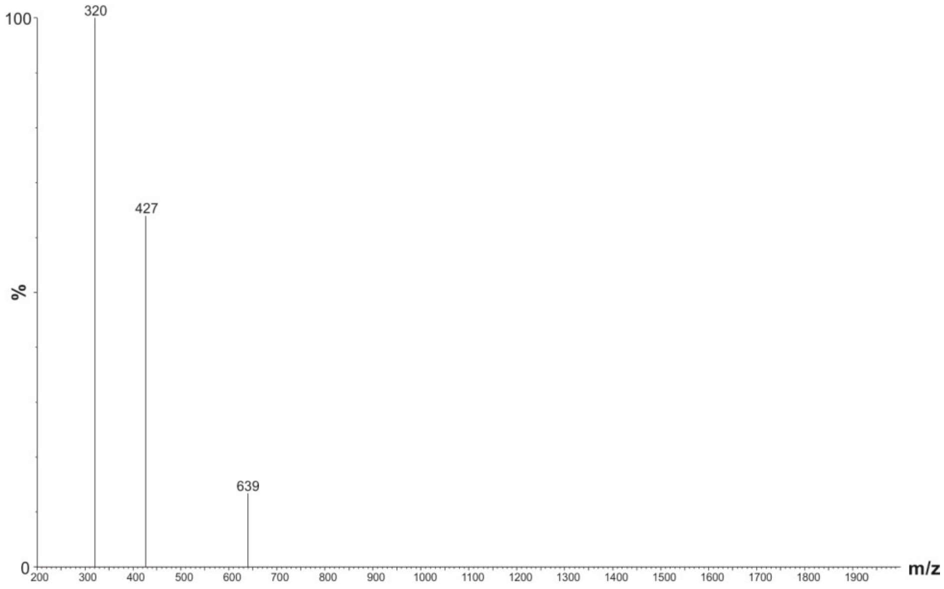


**Supplementary Figure 4. Analytical data sheet of Pam-3.**

Here we show the detailed analysis of purity and characterization of Pam-3.

**Supplementary Table 1. Antibiogram of ESKAPE pathogens used in this study.**

Bacteria susceptible to all (blue color) or intermediate/resistant to at least one (red color) of antibiotics per class following the EUCAST breakpoints. Black color is shown if the susceptibility to agents in that class is not assessed. Values are minimal inhibition concentrations (MIC) in µg/ml or for Chloramphenicol in diameter (mm).

| | *E. faecium* | | | *S. aureus* | | | *K. pneumoniae* | | |
|---|---|---|---|---|---|---|---|---|---|
| | DSM2918 | 11037 CHB | 20218 CHB | DSM20231 | ATCC33592 | ATCC43300 | 6727 | DSM30104 | 6970 |
| **Aminoglycosides** | | | | Gentamicin ≤ 0.5 | Gentamicin ≤ 0.5 | Gentamicin ≥ 16 | Gentamicin ≤ 1<br>Tobramycin ≤ 1 | Gentamicin ≤ 1<br>Tobramycin ≥ 16 | Gentamicin ≤ 1<br>Tobramycin ≥ 16 |
| **Anasamycins** | | | | Rifampicin ≤ 0.5 | Rifampicin ≥ 32 | Rifampicin ≤ 0.5 | | | |
| **Carbapenems** | Imipenem ≥ 16 | Imipenem ≥ 16 | Imipenem ≥ 16 | | | | Imipenem 2<br>Meropenem 2 | Imipenem ≤ 0.25<br>Meropenem ≤ 0.25 | Imipenem ≥ 16<br>Meropenem ≥ 16 |
| **Cephalosprins** | | | | | | | Cefuroxim ≥ 32<br>Ceftazidim 4 | Cefuroxim ≥ 64<br>Ceftazidim 16 | Cefuroxim ≥ 64<br>Ceftazidim 16 |
| **Fluoroquinolones** | Ciprofloxacin ≥ 8 | Ciprofloxacin ≥ 8 | Ciprofloxacin ≥ 8 | Ciprofloxacin ≥ 8 | Ciprofloxacin ≤ 0.5 | Ciprofloxacin ≤ 0.5 | Ciprofloxacin ≤ 0.25 | Ciprofloxacin ≥ 4 | Ciprofloxacin ≥ 4 |
| **Glycopeptides** | Vancomycin ≥ 32<br>Teicoplanin ≥ 32 | Vancomycin ≥ 32 | Vancomycin ≥ 32 | Vancomycin 1<br>Teicoplanin 0.75 | Vancomycin 1 | Vancomycin 1 | | | |
| **Glycylcyclines** | Tigecycline ≤ 0.12 | Tigecycline ≤ 0.12 | Tigecycline ≤ 0.12 | Tigecycline ≤ 0.12 | Tigecycline 0.5 | Tigecycline ≤ 0.12 | Tigecycline ≤ 0.5 | Tigecycline 4 | Tigecycline 4 |
| **Lincosamides** | | | | Clindamycin ≤ 0.25 | Clindamycin ≤ 0.5 | Clindamycin ≥ 8 | | | |
| **Lipopeptides** | Daptomycin 4 | | | Daptomycin 0.5 | Daptomycin 0.5 | Daptomycin 0.25 | | | |
| **Marcrolides** | | | | Erythromycin ≥ 8 | Erythromycin ≥ 8 | Erythromycin ≥ 8 | | | |
| **Monobactames** | | | | | | | | | |
| **Oxazolidinones** | Linezolid 2 | Linezolid 2 | Linezolid 1 | Linezolid 2 | Linezolid 2 | Linezolid 2 | | | |
| **Penicillins** | Ampicillin ≥ 32<br>Augmentin ≥ 256 | Ampicillin ≥ 32 | Ampicillin ≥ 32 | Oxacillin ≥ 4<br>Augmentin ≥ 256 | Oxacillin ≥ 4 | Oxacillin ≥ 4 | Ampicillin ≥ 32 | Ampicillin ≥ 32 | Ampicillin ≥ 32 |
| **Phenicols** | | | | Chloramphenicol 22mm | | | | | |
| **Polymyxins** | | | | | | | | Colistin 0.25 | |
| **Sulfonamides** | Trimethoprim/Sulfamethoxazol ≥ 320 | Trimethoprim/Sulfamethoxazol 160 | Trimethoprim/Sulfamethoxazol 20 | Trimethoprim/Sulfamethoxazol ≤ 10 | Trimethoprim/Sulfamethoxazol ≤ 10 | Trimethoprim/Sulfamethoxazol ≤ 10 | Trimethoprim/Sulfamethoxazol ≥ 320 | Trimethoprim/Sulfamethoxazol ≥ 320 | Trimethoprim/Sulfamethoxazol ≥ 320 |
| **Tetracyclins** | | | | Tetracyclin ≤ 1 | Tetracyclin ≥ 16 | Tetracyclin ≤ 1 | | | |

| | *A. baumannii* | | | *P. aeruginosa* | | | *E. coli* | | |
|---|---|---|---|---|---|---|---|---|---|
| | DSM30007 | ECII | LMG944 | DSM1117 | ATCC27853 | NRZ01677 | 6940 | DSM682 | DSM8695 |
| **Aminoglycosides** | Gentamicin ≥ 16<br>Tobramycin ≥ 16 | Gentamicin ≥ 16<br>Tobramycin ≥ 16 | Gentamicin ≤ 1<br>Tobramycin ≤ 1 | Gentamicin ≤ 1<br>Tobramycin ≥ 16 | Gentamicin ≥ 16<br>Tobramycin ≥ 16 | Gentamicin ≥ 16<br>Tobramycin ≥ 16 | Gentamicin ≤ 1<br>Tobramycin ≤ 1 | Gentamicin ≥ 16<br>Tobramycin ≥ 16 | Gentamicin ≤ 1<br>Tobramycin ≤ 1 |
| **Anasamycins** | | | | | | | | | |
| **Carbapenems** | Imipenem ≥ 16<br>Meropenem ≥ 16 | Imipenem ≥ 16<br>Meropenem ≥ 16 | Imipenem ≤ 0.25<br>Meropenem ≤ 0.25 | Imipenem ≥ 16<br>Meropenem 8 | Imipenem ≥ 16<br>Meropenem ≥ 16 | Imipenem ≥ 16<br>Meropenem ≥ 16 | Imipenem ≤ 0.25<br>Meropenem ≤ 0.25 | Imipenem 2<br>Meropenem 8 | Imipenem ≤ 0.25<br>Meropenem ≤ 0.25 |
| **Cephalosprins** | Ceftazidim ≥ 64 | Ceftazidim ≥ 64 | Ceftazidim ≤ 1 | Ceftazidim 16 | Ceftazidim ≥ 64 | Ceftazidim ≥ 64 | Cefuroxim ≥ 64<br>Ceftazidim 16 | Cefuroxim ≥ 64<br>Ceftazidim ≥ 64 | Ceftazidim ≤ 1 |
| **Fluoroquinolones** | Ciprofloxacin ≥ 4 | Ciprofloxacin ≥ 4 | Ciprofloxacin ≤ 0.25 | Ciprofloxacin ≥ 4 | Ciprofloxacin ≥ 4 | Ciprofloxacin ≥ 4 | Ciprofloxacin ≤ 0.25 | Ciprofloxacin ≥ 4 | Ciprofloxacin ≤ 0.25 |
| **Glycopeptides** | | | | | | | | | |
| **Glycylcyclines** | Tigecycline 4 | Tigecycline 4 | Tigecycline ≤ 0.5 | | | | Tigecycline ≤ 0.5 | Tigecycline ≤ 0.5 | Tigecycline ≤ 0.5 |
| **Lincosamides** | | | | | | | | | |
| **Lipopeptides** | | | | | | | | | |
| **Marcrolides** | | | | | | | | | |
| **Monobactames** | | | | Aztreonam > 256 | | | | | |
| **Oxazolidinones** | | | | | | | | | |
| **Penicillins** | Ampicillin/Sulbactam ≥ 32 | Ampicillin/Sulbactam ≥ 32 | Ampicillin/Sulbactam ≤ 2 | Piperacillin ≥ 128 | Piperacillin ≥ 128 | Piperacillin ≥ 128 | Ampicillin ≥ 32<br>Piperacillin ≥ 128 | Ampicillin ≥ 32<br>Piperacillin ≥ 128 | Ampicillin 8<br>Piperacillin ≤ 4 |
| **Phenicols** | | | | | | | | | |
| **Polymyxins** | Colistin 16 | | | Colistin 1 | | | | | |
| **Sulfonamides** | Trimethoprim/Sulfamethoxazol ≤ 20 | Trimethoprim/Sulfamethoxazol ≤ 20 | Trimethoprim/Sulfamethoxazol ≤ 20 | | | | Trimethoprim/Sulfamethoxazol ≥ 320 | Trimethoprim/Sulfamethoxazol ≥ 320 | Trimethoprim/Sulfamethoxazol ≤ 20 |
| **Tetracyclins** | Tetracyclin ≥ 256 | | | | | | | | |
