# Supplementary material for: Curbing gastrointestinal infections by defensin fragment modifications without harming commensal microbiota
Source: Commun Biol. 2021 Jan 8;4:47. doi: 10.1038/s42003-020-01582-0 (PMC7794397; doi:10.1038/s42003-020-01582-0)
Supplement: Supplementary file 3 — Description of Supplementary Files [file 42003_2020_1582_MOESM3_ESM.pdf]

Description of Additional Supplementary Files

File name: Supplementary Data 1

Description: Source data for figure 1b, figure 2b, figure 3, figure 4, figure 6 and supplementary figure 1
