# Supplementary material for: Curbing gastrointestinal infections by defensin fragment modifications without harming commensal microbiota
Source: Commun Biol. 2021 Jan 8;4:47. doi: 10.1038/s42003-020-01582-0 (PMC7794397; doi:10.1038/s42003-020-01582-0)
Supplement: Supplementary file 5 — Reporting Summary [file 42003_2020_1582_MOESM5_ESM.pdf]

natureresearch

Corresponding author(s): Louis Koeninger
E-mail: Louis.koeninger@med.uni-tuebingen.de

Last updated by author(s): Nov 13, 2020

Reporting Summary

Nature Research wishes to improve the reproducibility of the work that we publish. This form provides structure for consistency and transparency in reporting. For further information on Nature Research policies, see Authors & Referees and the Editorial Policy Checklist .

Statistics

For all statistical analyses, confirm that the following items are present in the figure legend, table legend, main text, or Methods section.

n/a	Confirmed	
☐	☒	The exact sample size (*n*) for each experimental group/condition, given as a discrete number and unit of measurement
☐	☒	A statement on whether measurements were taken from distinct samples or whether the same sample was measured repeatedly
☐	☒	The statistical test(s) used AND whether they are one- or two-sided<br>*Only common tests should be described solely by name; describe more complex techniques in the Methods section.*
☐	☒	A description of all covariates tested
☐	☒	A description of any assumptions or corrections, such as tests of normality and adjustment for multiple comparisons
☐	☒	A full description of the statistical parameters including central tendency (e.g. means) or other basic estimates (e.g. regression coefficient) AND variation (e.g. standard deviation) or associated estimates of uncertainty (e.g. confidence intervals)
☐	☒	For null hypothesis testing, the test statistic (e.g. *F*, *t*, *r*) with confidence intervals, effect sizes, degrees of freedom and *P* value noted<br>*Give P values as exact values whenever suitable.*
☐	☒	For Bayesian analysis, information on the choice of priors and Markov chain Monte Carlo settings
☐	☒	For hierarchical and complex designs, identification of the appropriate level for tests and full reporting of outcomes
☐	☒	Estimates of effect sizes (e.g. Cohen's *d*, Pearson's *r*), indicating how they were calculated

Our web collection on statistics for biologists contains articles on many of the points above.

Software and code

Policy information about availability of computer code

Data collection	Detailed bioinformatics pipeline is described in the manuscript (materialand methods section). Fluorescence microscopy was carried out using a Zeiss Axio Observer Z1 automated microscope. Images were acquired with an Orca Flash 4.0 V2 camera (Hamamatsu), C Plan-Apo 63x/1.4 Oil DIC and alpha Plan-Apochromat 100x/1.46 Oil Ph3 objectives (Zeiss) and processed using the Zen software package (Zeiss). TEM was carried out using a a Tecnai Spirit and SEM with a Hitachi Regulus 8230 field emission scanning electron microscope.
Data analysis	GraphPad Prism8, Usearch8.1 software package (http://www.drive5.com/usearch/), UPARSE algorithm, Silva database v128, RDP Classifier executed at 80% bootstrap confidence, R statistical programming environment package phyloseq and ggplot2. Custom R code will be deposited on Github (https://github.com/strowig-lab)

For manuscripts utilizing custom algorithms or software that are central to the research but not yet described in published literature, software must be made available to editors/reviewers. We strongly encourage code deposition in a community repository (e.g. GitHub). See the Nature Research guidelines for submitting code & software for further information.

Data

Policy information about availability of data

All manuscripts must include a data availability statement. This statement should provide the following information, where applicable:

- Accession codes, unique identifiers, or web links for publicly available datasets
- A list of figures that have associated raw data
- A description of any restrictions on data availability

Data that support the findings of this study are stored in excel file format and are available from the corresponding author upon reasonable request. 16S rRNA gene sequencing data have been deposited in the NCBI (Bioproject Database) under the accession number: PRJEB37278.

October 2018

Field-specific reporting

Please select the one below that is the best fit for your research. If you are not sure, read the appropriate sections before making your selection.

☒ Life sciences ☐ Behavioural & social sciences ☐ Ecological, evolutionary & environmental sciences

For a reference copy of the document with all sections, see nature.com/documents/nr-reporting-summary-flat.pdf

Life sciences study design

All studies must disclose on these points even when the disclosure is negative.

Sample size	Sample-size calculation was not initially performed for in vitro experiments but a sufficient number of replicates were taken based on literature standards, prior experience with these types of data, and analysis of the current data, which indicates the reproducibility of the data.
Data exclusions	No data were excluded from the study.
Replication	Experiments were repeated at least 3 times to confirm reproducibility. In all the cases the experiments were successfully replicated
Randomization	Mice were randomly distributed among the cages. Further randomization was not performed.
Blinding	The investigators were not blinded to group allocation.

Reporting for specific materials, systems and methods

We require information from authors about some types of materials, experimental systems and methods used in many studies. Here, indicate whether each material, system or method listed is relevant to your study. If you are not sure if a list item applies to your research, read the appropriate section before selecting a response.

Materials & experimental systems

n/a	Involved in the study	
☒	☐	Antibodies
☒	☐	Eukaryotic cell lines
☒	☐	Palaeontology
☐	☒	Animals and other organisms
☒	☐	Human research participants
☒	☐	Clinical data

Methods

n/a	Involved in the study	
☒	☐	ChIP-seq
☒	☐	Flow cytometry
☒	☐	MRI-based neuroimaging

Animals and other organisms

Policy information about studies involving animals; ARRIVE guidelines recommended for reporting animal research

Laboratory animals	C57BL/6N mice were generated and maintained (including breeding and housing) at the animal facilities of the Helmholtz Centre for Infection Research (HZI) under enhanced specific pathogen-free (SPF) conditions. Animals used in the experiments were gender and age matched. Female and male mice with an age of 8-12 weeks were used.
Wild animals	This study did not involve wild animals.
Field-collected samples	This study did not involve samples collected from the field.
Ethics oversight	All animal experiments were performed in agreement with the local government of Lower Saxony, Germany (approved permission No. 33.19-42502-04-18/2499).

Note that full information on the approval of the study protocol must also be provided in the manuscript.
